# The effect of a fennel seed extract on the STAT signaling and intestinal barrier function

**DOI:** 10.1371/journal.pone.0271045

**Published:** 2022-07-08

**Authors:** Barun Das, John Rabalais, Philip Kozan, Tina Lu, Nassim Durali, Kevin Okamoto, Matthew D. McGeough, Beom Jae Lee, Kim E. Barrett, Ronald Marchelletta, Mamata Sivagnanam

**Affiliations:** 1 Department of Pediatrics, University of California, San Diego, La Jolla, CA, United States of America; 2 Department of Medicine, University of California, San Diego, La Jolla, CA, United States of America; 3 Department of Gastroenterology, Korea University, Guro Hospital, Seoul, South Korea; 4 Department of Physiology and Membrane Biology, University of California, Davis, Davis, CA, United States of America; 5 Rady Children’s Hospital, San Diego, CA, United States of America; Indiana University School of Medicine, UNITED STATES

## Abstract

**Background:**

*Foeniculum vulgare*, *F*. *vulgare*, commonly known as fennel, is believed to be one of the world’s oldest medicinal herbs and has been exploited by people for centuries as a nutritional aid for digestive disorders. In many southeast Asian countries, it is ingested as an after-meal snack, mukhvas, due to its breath-freshening and digestive aid properties. *F*. *vulgare* is used in some countries, such as Iran, as a complementary and alternative treatment for inflammatory bowel disease (IBD).

**Methods:**

This study investigated the effects of fennel seed extract on intestinal epithelium barrier function and the Signal Transducer and Activator of Transcription (STAT) pathway. This pathway is active in inflammatory bowel disease. To study the protective effects of fennel seed extract *in vitro*, monolayers derived from the T84 colonic cell line were challenged with interferon-gamma (IFN-γ) and monitored with and without fennel seed extract. To complement our *in vitro* studies, the dextran sodium sulfate induced murine colitis model was employed to ascertain whether the protective effect of fennel seed extract can be recapitulated *in vivo*.

**Results:**

Fennel seed extract was shown to exert a protective effect on transepithelial electrical resistance (TEER) in both T84 and murine models and showed increases in tight junction-associated mRNA in T84 cell monolayers. Both models demonstrated significant decreases in phosphorylated STAT1 (pSTAT1), indicating reduced activation of the STAT pathway. Additionally, mice treated with fennel seed showed significantly lower ulcer indices than control mice.

**Conclusions:**

We conclude barrier function of the gastrointestinal tract is improved by fennel seed extract, suggesting the potential utility of this agent as an alternative or adjunctive therapy in IBD.

## Introduction

*Foeniculum vulgare* (*F*. *vulgare*), commonly known as fennel, is a flowering plant species in the family Apiaceae. It is believed to be one of the world’s oldest medicinal herbs and has been exploited by people for centuries for its reported anti-inflammatory and antipathogenic properties [1]. In many southeast Asian countries it is ingested as an after-meal snack, mukhvas, due to its breath-freshening and digestive aid properties.

There is a rising interest in herbal therapies for IBD worldwide, with clinical studies being done on a variety of natural products, such as *aloe vera* gel and *Andrographis paniculate* extract, which proved effective compared to placebos [2]. Previous studies have examined various products derived from *F*. *vulgare*, though not directly in the intestine. One study showed that oil extracted from *F*. *vulgare* was characterized to have safe antithrombotic activity through its broad spectrum antiplatelet activity, clot destabilizing effect, and vasorelaxant action [3]. This study also demonstrated that fennel oil provided significant protection from ethanol-induced gastric lesions in rats. Fennel oil extract has been shown to have *in vitro* antifungal activity when column fractions are screened against MDR strains of *Mycobacterium tuberculosis* [4]. Anethole, a major component of fennel oil, is an organic compound that is widely used as a flavoring substance and also has been shown to have anti-proliferative effects on prostate cancer cells [5]. It is a derivative of phenylpropene, a type of aromatic compound that occurs widely in nature. In addition to the beneficial effects of fennel oil, fennel “waste” (components remaining after oil extraction) has been studied and found to exhibit high antioxidant activity [6, 7]. *F*. *vulgare* is also used in some countries, such as Iran, as a complementary and alternative treatment for inflammatory bowel disease [1, 8, 9]. Additionally, a combination of *F*. *vulgare* and turmeric oils has been shown to increase quality of life in IBS patients, highlighting the wide-ranging benefits of *F*. *vulgare* on the body, particularly in the gastrointestinal system [10]. Other herbal therapy studies have shown far reaching effects in the context of IBD, with data suggesting herbal remedies are able to mediate damage to barrier function, immune response, and even restore gut microbiota [11]. This study specifically investigates the use of *fennel seed extract* in mediating mechanisms of inflammatory bowel disease.

Inflammatory bowel disease affects nearly 1 million people annually in the United States [12]. IBD is characterized by chronic inflammation of the small and/or large intestines. The pathogenesis of IBD involves a multitude of genetic and environmental factors that are presumed to cause an excessive and inappropriate mucosal immune response [12–14]. In IBD, the balance between pro- and anti-inflammatory mediators is shifted, leading to infiltration of the lamina propria with immune cells that release pro-inflammatory cytokines such as interferon-gamma (IFN-γ). To ensure intestinal homeostasis, a robust barrier between epithelial cells is essential to protect against foreign antigens. The barrier formed by epithelial cells is partly comprised of the apically-located tight junction complex, which includes occludin (OCLD), claudins, and tight junction protein-1 (TJP-1) [15]. In IBD, this barrier becomes compromised [16]. The cytokines present in IBD damage the intestinal barrier, resulting in clinical and pathologic manifestations including mucosal friability, decreased tissue resistance, and increased paracellular permeability [17].

IFN-γ is known to signal through the Janus Kinase (JAK)/ Signal Transducer and Activator of Transcription (STAT) pathway, and activation of this pathway, including phosphorylation of STAT1, has been demonstrated in IBD [18, 19]. The pathway is initiated when IFN-γ binds to its receptor and induces dimerization of its subunits. The JAK dimer is then recruited to the receptor and becomes activated, phosphorylating STAT proteins [20–22]. *In vitro* and *in vivo* studies show that activated IFN-γ receptor can modify epithelial barrier function by mechanisms that include new protein synthesis, membrane trafficking, kinase activation, cytoskeletal modulation and epithelial apoptosis [23]. Aberrant activation of IFN-γ receptor induces epithelial dysfunction with pathology similar to that observed in Crohn’s disease [18, 24, 25]. Of interest, the JAK/STAT pathway is targeted by Tofacitinib, a novel small-molecule drug investigated for its ability to inhibit the JAK family in the setting of IBD. This results in immunosuppression through downregulation of inflammatory molecules [26, 27]. Previous data have shown that components of *F*. *vulgare* oil extract potently inhibit TNF-induced activation of NF-κB in B cells [28]. and also inhibit the release of interleukin-1β following administration of LPS to rats [29]. Based on the known impact of *F*. *vulgare* on responses evoked by other inflammatory mediators, we hypothesized that *F*. *vulgare* seed extract may also play a role in modulating the JAK/STAT pathway. The role of *F*. *vulgare* seed extract on intestinal barrier function and the JAK/STAT pathway has yet to be studied and could provide an additional mechanism underlying fennel*’s* anti-inflammatory properties and its use as a complementary treatment for IBD. In this study, we utilized the T84 cell line to examine any protective effects of fennel seed extract in cells treated with IFN-γ. This colonic adenocarcinoma cell line has been widely used as a model for studies of epithelial barrier function [30]. To understand how *F*. *vulgare* seed extract may ameliorate IBD, a murine model of dextran sodium sulfate (DSS)-induced colitis was employed [31].

## Methods

### Ethics approval

Animal experiments were conducted per international guidelines. The protocols of this animal research study were approved by the UCSD IACUC. No animals died during the experiment, except by humane euthanasia.

### Fennel seed extract

Fennel seed extract was obtained from Nature’s Answer (Hauppauge, NY) and was produced by crude extraction from dry *F*. *vulgare* seeds by the company. Ingredients found in the extract include: Fennel seed extract, 15% Certified Organic Ethyl Alcohol, water, and Vegetable Glycerin. Nature’s Answer performs DNA based quality control to ensure the presence of fennel seed within the extract in addition to other appropriate quality control processes recommended by the FDA.

### Cell culture

T84 cells were cultured in 1:1 Dulbecco’s modified Eagle’s Medium/F-12 Ham’s medium with 15 mM L-glutamine (Mediatech Inc., Manassas, VA), 5% bovine calf serum (Invitrogen, Grand Island, NY), and 1% penicillin-streptomycin (Mediatech Inc.) and maintained according to a standard protocol [32]. Cells were divided into 6 groups: control (receiving ethanol as vehicle), IFN (100 U/mL IFN-γ), and fennel seed extract /IFN treatments with 4 increasing dosages of fennel seed extract (4, 6, 7.5 and 9 μL/mL) with IFN (100 U/mL IFN-γ). The equivalent concentration of ethanol that matched the concentration of ethanol present in the highest fennel seed extract (9 μL/mL) condition was used as the vehicle control for all experiments. Fennel seed extract treatments began 2 days after culturing on 24 well plates and IFN was added 2 days later. Groups were plated in triplicate at 0.5 x 10^6^ cells per well. RNA and protein were collected 72 hours after IFN treatment. To eliminate the concern of fennel seed extract toxicity on T84 cells, Different concentrations of fennel seed extract were diluted in cell media. For resistance studies, T84 cells were grown on semipermeable 12 mm Millicell-HA cell culture inserts with 0.5x10^6^ cells added per insert. Cells were maintained in media as above. Media was changed every 2 days until a stable monolayer was established, approximately 10 days [32]. Epithelial health was monitored using barrier function as a readout measured by transepithelial electrical resistance (TEER) [33].

### Cell viability analysis

Cell viability of the T84 cells was assessed with propidium iodide (PI) staining using flow cytometry in a similar way as described earlier [34] after treatment with fennel seed extract and vehicle (Ethanol). The single cell suspension of T84 cells was obtained after trypsinization for 5 minutes at 37°C followed by mechanical disruption and passing through a 70 micron cell strainer (Corning #352235, Corning, NY) to separate cell clumps. The cells were resuspended in PBS and PI (Cat# P4864, Sigma-Aldrich, St Louis, MO) was added to the cell suspension at 0.5 μg/mL. After incubation for 15 minutes in the dark, the cells were acquired in BD Accuri™ C6 (BD, Franklin lakes, NJ) Flowcytometer. The percentage of PI positive cells was analyzed with FlowJo software (FlowJo LLC, Ashland, OR).

A vehicle treated group was also included and compared with an untreated control in the experiment to assess any negative effects of ethanol. Forward scatter (FSC)-Area (A) vs FSC-Height (H) pulse gating strategy was incorporated to exclude the duplets and cell aggregates from the total cell population as described earlier [34]. The PI positive staining in the gated population was calculated after normalizing the mean fluorescent intensity of the respective unstained population.

### Murine colitis studies

C57BL/6 mice obtained from Jackson Labs (Bar Harbor, ME) were used for all *in vivo* studies. Mice weighed 20–25 grams and were housed in plastic cages with flake bedding. Mice were randomized to 4 groups. All mouse groups were orally gavaged (150 μL) daily for 8 days with water (control and DSS group), or fennel seed extract dissolved in drinking water at 4.5 uL/mL (fennel seed extract alone, DSS/ fennel seed extract), a concentration used in previous murine studies during the daytime [3, 35]. DSS and DSS/ fennel seed extract groups received 3.5% DSS in their drinking water for 5 days followed by 3 days of normal drinking water in their group cages. Mice had access to chow and water and rooms were kept on light dark cycle. Mice were euthanized per UCSD protocol using carbon dioxide inhalation. All studies were approved by the UCSD Institutional Animal Care and Use Committee and no adverse events were noted. Upon sacrifice of mice, gross health of colonic tissue (friability) and stool characteristics, such as presence of blood, color, softness, etc., were noted.

### Electrical resistance

To assess monolayer integrity, TEER was measured across T84 monolayers with a voltohmeter (World Precision Instruments, Sarasota, FL). The monolayer was considered mature when the TEER of the monolayer reached a stable value of approximately 1000 ohms.cm^2^. In fennel seed extract pre-treatment studies, fennel seed extract or vehicle was added to both apical and basolateral media to the cells in the transwell upon reaching this baseline. TEER values continued to be taken for each of the groups. After 2 days, IFN-γ was added basolaterally to the IFN and fennel seed extract groups and TEER values were followed for 5 days. In fennel seed extract rescue studies, cells were plated in the same manner as pre-treatment studies. Upon reaching maturity, either IFN-γ or vehicle was given to corresponding groups and fennel seed extract was added to monolayers 2 days post IFN-γ or vehicle addition.

For murine studies, animals were sacrificed by carbon dioxide exposure per institutional guidelines and full-thickness segments of mid colon were mounted in Ussing chambers (Physiological Instruments, San Diego, CA) per protocol (mouse tissue window area: 0.09 cm^2^) [36]. Colonic tissue was excised and washed in Ringer’s solution containing 140 mM Na^+^, 5.2 mM K^+^, 1.2 mM Ca^2+^, 0.8 mM Mg^+^, 120 mM Cl^-^, 25 mM HCO_3_^-^, 2.4 mM H_2_PO_4_^-^, 0.4 mM HPO_4_^2-^, 10 mM glucose. Tissue was then mounted into the slides and bathed in an oxygenated Ringer’s solution at 37°C that had been previously equilibrated in the Ussing chambers for 30 minutes. A transepithelial current pulse of 1 μA was administered through the chamber and the resulting voltage drop between chambers was measured to assess tissue viability and TEER was calculated from the resulting voltage deflection using Ohm’s law (V = IR).

### Western blotting

T84 cells were suspended in RIPA lysis buffer and proteins were extracted and analyzed according to a published protocol [37]. Samples were resuspended in loading buffer (50 mM Tris (pH 6.8), 2% SDS, 100 mM dithiothreitol, 0.2% bromphenol blue, and 20% glycerol).

Mid colon samples were placed in nonyl phenoxypolyethoxyethanol-40 (NP-40) buffer containing 0.9% NaCl, 10% glycerol, 50 mM Tris (pH 2.8), 0.1% NP40, 5 mM EDTA, 20 μM NaF, 1 μg/ml antipain, 1 μg/ml pepstatin, 1 μg/ml leupeptin, 1 mM NaVO_3_, and 100 μg/ml phenylmethylsulfonyl fluoride and lysed using a mini bead beater (BioSpec Products, Bartlesville, OK). The lysate was centrifuged and the supernatant containing the proteins was removed [37]. Samples were suspended in loading buffer as above.

Cell and tissue lysates were diluted at a 1 to 1 ratio of lysate to loading buffer and loaded onto Mini-Protean TGX precast gels (BioRad), electrophoresed, then transferred onto PVDF membranes. Membranes were blocked with 5% bovine serum albumin/TBST.

Immunostaining of the blots was performed using rabbit antibodies to pSTAT1 and STAT1 (Cell Signaling Technologies, Beverly, MA) at 1:1000. A mouse monoclonal antibody to β-actin at 1:5000 (Sigma) was used to correct for loading. Horseradish peroxidase-conjugated anti-mouse or anti-rabbit IgG secondary antibodies (Cell Signaling Technologies, Beverly, MA) were used at 1:2000 dilutions. A semiquantitative measurement of band density was performed using ImageJ for Windows software.

### Q-PCR

Total RNA from T84 cells was isolated using Direct-zol RNA MiniPrep kits (Zymo, Irvine, California). RNA was extracted initially using TRIzol (Invitrogen, Carlsbad, California). First strand cDNA was synthesized with iScript cDNA Synthesis kit (Bio Rad, Irvine, California) using the recommended protocol. Real time PCR reactions were set up using SYBR™ Green Master Mix (Applied Biosystems™) and thermal cycling performed on a StepOnePlus Real-Time PCR System using Step One software v2.0. (Applied Biosystems, Carlsbad, CA) with the following conditions (95°C for 10 min, followed by 95°C for 15 s, 60°C for 1 min for 40 cycles), Primers were obtained from IDT (Integrated DNA Technologies, Coralville, IA). Glyceraldehyde 3-phosphate dehydrogenase (GAPDH) was used as the housekeeping gene. STAT-1 and GAPDH primers were diluted to 100μM. TJP-1 and OCLD primers were diluted to 50μM.

### Tissue histologic imaging

Segments of mid colon were excised and fixed with 4% paraformaldehyde for 24 hours at room temperature, then paraffin-embedded and sectioned onto glass slides. De-paraffination was performed and samples were heated in 10mM sodium citrate buffer. Hemotoxylin and eosin staining was performed by the Allergy Institute of La Jolla [38]. Imaging was performed using a Leica DMi1 inverted microscope at 20x magnification using LAS 4.10 EZ acquisition software.

### Ulcer indices

To assess the effect of fennel seed extract on intestinal structural integrity, ulcer indices were assigned on a scale of 0 to 19 based on an established scoring system assessing inflammation on tissue sections [39] by four reviewers blinded to the experimental condition. Criteria for assessment of colonic inflammation and ulceration were as follows:

- Inflammation: 0 –Normal, 1- Minimal infiltration of lamina propria, focal to multifocal, 2 –Mild infiltration of lamina propria, multifocal, mild gland separation, 3 –Moderate to mixed infiltration, multifocal with minimal edema, 4 –Marked mixed infiltration into submucosa and lamina propria with extensive areas of gland separation, enlarged Peyer’s patches, edema
- Epithelium: 0 –Normal, 1 –Minimal damage, focal mucosal hyperplasia, 2 –Mild damage, multifocal tufting of rafts of epithelial cells with increased numbers of goblet cells, 3 –Moderate damage, extensive local or multifocal erosion or epithelial attenuation, 4 –Marked locally extensive mucosal ulceration
- Glands: 0 –Normal, 1 –Rare gland dilation present, 2 –multifocal gland dilation, 3—multifocal gland dilation with abscessation and occasional loss of glands, 4 –locally extensive to subtotal loss of glands
- Depth of Lesion: 0 –None, 1 –Mucosa, 2 –Mucosa and submucosa, 3 –Transmural
- Extent of Section Affected: 0 –None, 1 –Minimal < 10%, 2 –Mild 10–25%, 3 –Moderate 26–50%, 4 –Severe > 50%

Each of the criteria were given separate scores and summed to give an overall index score per image. Four mice were present in each treatment condition (Vehicle, FN, DSS, FN/DSS). Four random images were taken from each mouse and provided to the reviewers for blinded scoring. The average ulcer index score for each mouse was generated.

### Immunofluorescence staining

In order to evaluate the expression and localization of tight junction proteins, immunofluorescent staining was performed in T84 cells. T84 cells were cultured in 8 well chamber slides (Nunc™ Lab-Tek™ II) and treatments, as stated above, were added in the culture media along with untreated control. At the end of experimental period, the cells were washed twice with PBS and fixed with ice cold acetone: methanol (1:1) for 15 minutes in -20 ^ο^C freezer. After aspirating the fixative and washing with PBS, the specimens were incubated in blocking buffer (with 3% goat serum in PBS) for 1 hour at room temperature, The primary antibodies (TJP-1 (Cat# 21773-1-AP.Proteintech) and Occludin Cat# 27260-1-AP Proteintech) were prepared in blocking buffer at 1:200 dilution and then incubated with the cells in chamber slides for overnight in 4^ο^C. After washing with PBS, the cells were incubated with Alexa Fluor 488 goat anti rabbit (Invitrogen) secondary antibody, prepared in blocking buffer at 1:200 dilution for 1 hour at room temperature. Nuclei were stained using the Draq5 fluorescent probe (Thermo Fischer Scientific) in PBS and incubated for 10 minutes at room temperature. Finally, the samples were washed in PBS and mounted using Prolong Gold antifade reagent (Life Technologies, Eugene, OR). Slides were imaged using a Leica (Leica Microsystems, Buffalo Grove, IL) confocal imaging system (DMI4000 B) using a 25× Plan-Apo 0.8 numerical aperture with 40X objective lens. Images were captured using LASX v4.1 Image Acquisition software supplied by Leica.

### Statistical analysis

One-way and Two-way Anova with Tukey’s multiple comparison test were performed using GraphPad Prism version 8.00 for Windows (GraphPad Software, La Jolla, CA). All data were pre tested for normal distribution with Shapiro-Wilk test before analyzing with Anova. Data from all animal experiments were included in data analysis. All raw data used and analyzed during the current study are available from the corresponding author on request.

## Results

### Fennel seed extract has protective effects on barrier integrity in T84 cells

**Fig 1:**
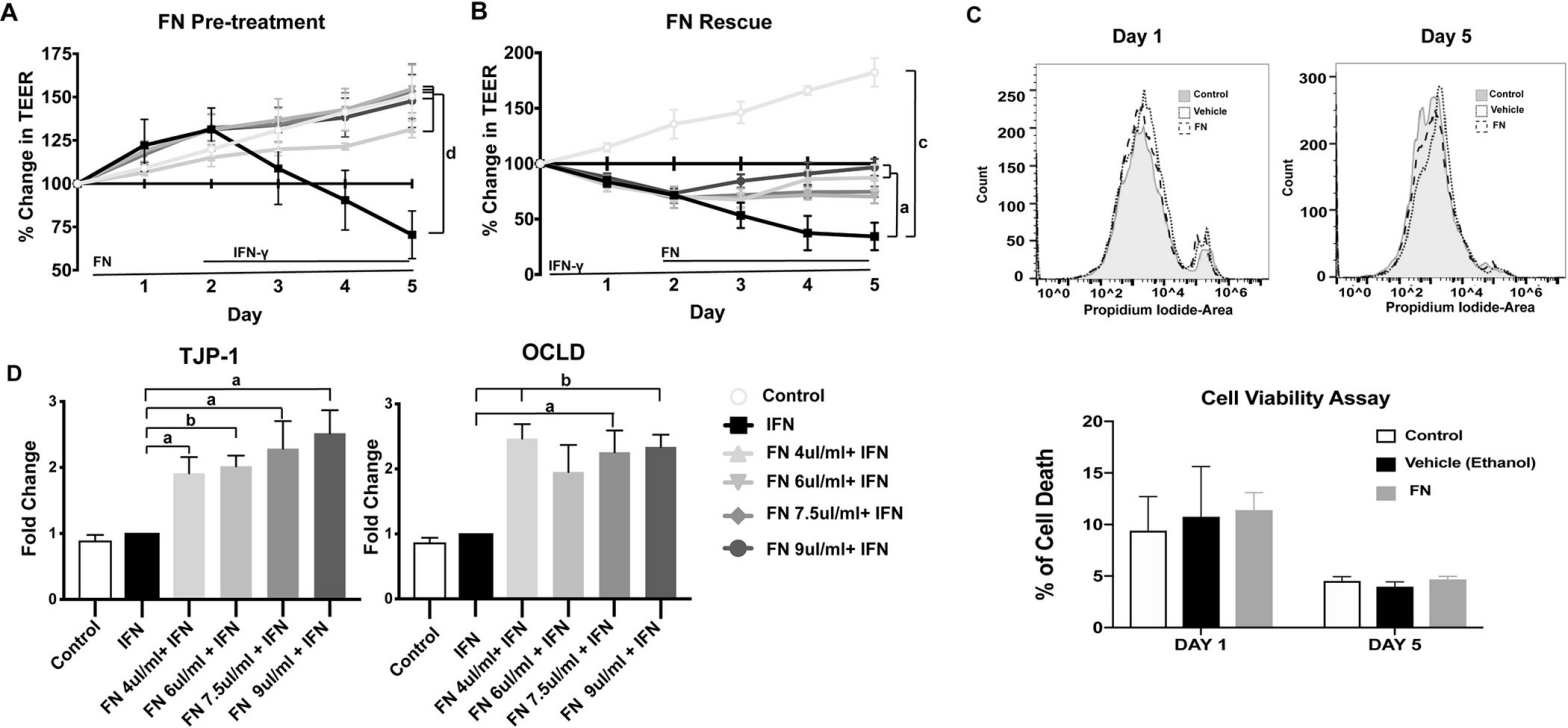
Fennel seed extract (FN) treatment of T84 cells improves tight junction functionality. A) Fennel seed extract (FN) pretreatment (with increasing dosage) of T84 cells treated with IFN-γ prevents decrease of TEER. B)) Fennel seed extract (FN) addition post IFN-γ treatment shows restorative effects on TEER. C) Propidium Iodide staining of Control, FN, and Vehicle treated T84 cells show no change in cell viability. D) Fennel seed extract (FN) treatments increase transcription of tight junction genes TJP-1 and OCLD. N = 4; a = P < 0.05; b = P < 0.01; c = P < 0.001; Data shown as mean + SD. Significance calculated against IFN-γ group in all experiments.

TEER correlates positively with epithelial barrier function [33]. As expected, addition of IFN-γ reduced the TEER of T84 cell monolayers day 4 onwards (p<0.001).when compared to vehicle treated control [40] However, in T84 cells that were pre-treated with fennel seed extract there was no change in TEER upon addition of IFN when compared with vehicle treated controls. All the fennel seed extract -treated cells group displayed a 30–50% increase TEER when compared to cells treated with IFN-γ alone over the course of the treatment, matching the trajectory of vehicle-treated control cells, indicating little to no adverse effects due to IFN-γ treatment (Fig 1A). In order to assess whether the fennel seed extract treatment has any detrimental effect on T84 cells, a cell viability assay was performed with PI staining using flow cytometry at day 1 and day 5 (maximum length of the experiments) of post treatment. The percentage of PI positive dead cells was found to be equivalent among non-treated group, vehicle and fennel treated group at both day 1 and day 5 post treatment. This result confirmed that both the fennel seed extract and ethanol vehicle had no detrimental effect on T84 cell viability (Fig 1C). In a separate experiment, fennel seed extract was administered to T84 monolayers after IFN-γ treatment. As before, IFN-γ addition immediately decreased TEER values, however upon addition of fennel seed extract, TEER values were stabilized, preventing further loss of barrier integrity, and in two cases being restored back to baseline levels (Fig 1B). By day 5 of the experiment, fennel seed extract at 4.5 and 9 μL/mL treatments showed a significant increase in TEER values compared to IFN-γ treated cells.

To further elucidate the beneficial effects of fennel seed extract on barrier function, expression of mRNA for selected tight junction proteins was evaluated via qRT-PCR. TJP-1 and OCLD were examined as a possible explanation for the ability of fennel *seed* extract to protect barrier function. There were significant increases in mRNA for both TJP-1 and OCLD in fennel seed extract*-*treated groups compared to the IFN-γ only treated groups (Fig 1D, P<0.05). TJP-1 transcripts increased with rising fennel seed extract dosages, with the highest concentration of fennel seed extract eliciting a 2.2 fold increase compared IFN-γ treated cells. There was also a significant increase in TJP-1 between fennel seed extract with 4.5 and 9 μL/mL treatments. OCLD transcripts were increased by fennel seed extract between 1.9 and 2.5 fold across all conditions. This effect was not dependent on the concentration of fennel seed extract, unlike TJP-1 expression.

**Fig 2:**
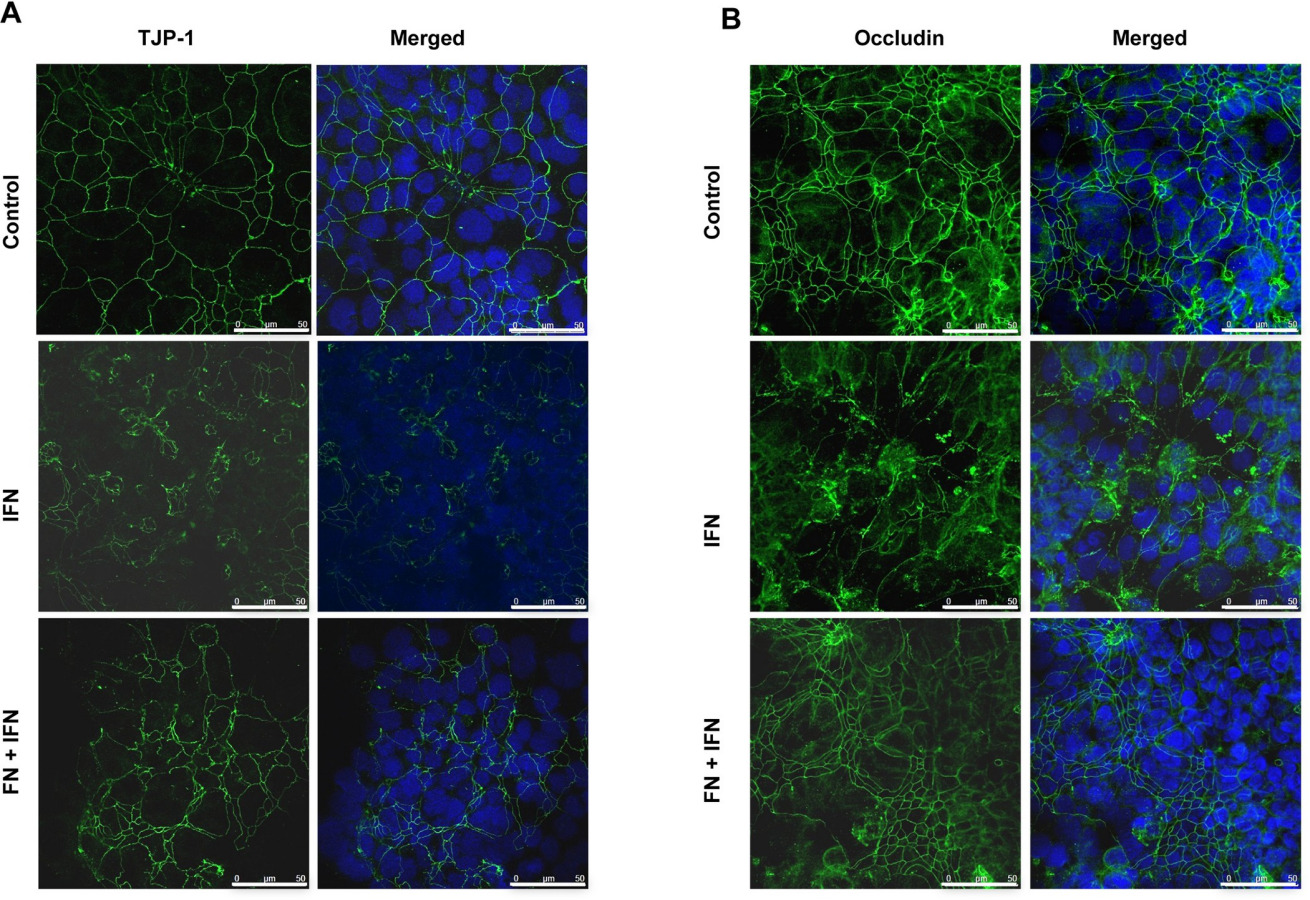
Fennel seed extract (FN) treatment (7.5 μl/ml) prevents the mislocalization and disruption of Tight junction protein 1 (TJP-1) (A) and Occludin (B) in IFN-γ treated T84 cells. Scale bar represents 50 μm. Representative confocal images from at least 5 independent experiments.

In order to investigate the distribution and expression of tight junction proteins upon treatment with fennel extract, TJP1 and occludin were immunostained in T84 cells and assessed with confocal microscopy. As expected, both the markers were expressed in the borders between the apical and basolateral cell surface domains in vehicle treated control cells (Fig 2). The distribution and expression of both the markers were altered in IFN-γ treated cells indicating the detrimental effect IFN-γ on tight junction proteins which contributes to the decreased TEER. Pretreatment with fennel extract prevented the mis-localization of the tight junction proteins from apical and basolateral cell surfaces upon IFN-γ treatment (Fig 2).

### Fennel seed extract attenuates activation of STAT1

**Fig 3:**
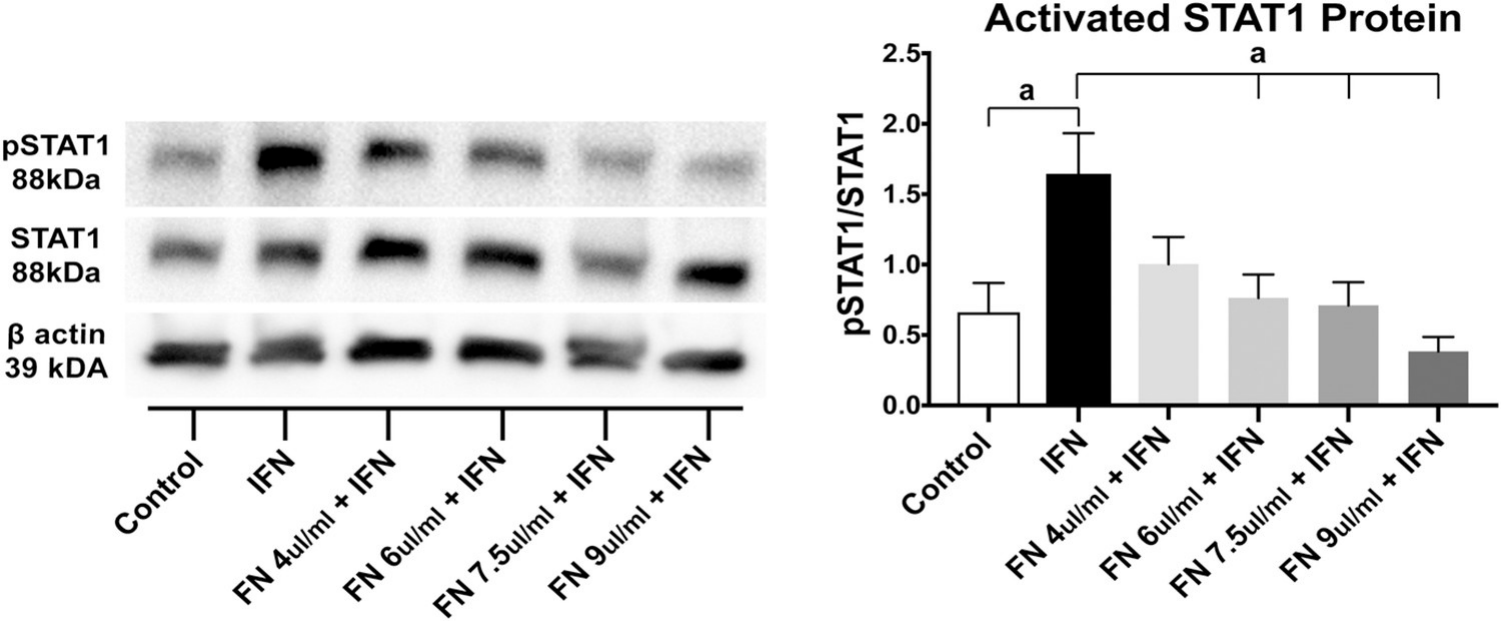
Fennel seed extract (FN) treated T84 cells show decreased STAT1 activation. Western blot analysis of pSTAT1 and STAT1 show decreasing pSTAT1 with increasing doses of fennel seed extract. N = 3; a = P < 0.05; b = P < 0.01; c = P < 0.001; FN 1-FN 4 correlate to increasing doses of fennel seed extract. Data shown as mean + SD. Significance calculated against IFN-γ group in all experiments.

STAT1 activation was examined to determine other possible effects of fennel seed extract on epithelial cells. Western blotting for activated phosphorylated STAT-1 (pSTAT-1) showed marked decreases in each of the of fennel seed extract treated groups, with the control group showing little pSTAT-1 as expected (Fig 3). Analysis of the ratio between pSTAT-1/STAT-1 showed a significant dose-dependent reduction in the pSTAT-1/STAT-1 ratio in response to fennel seed extract. The lowest dosage of fennel seed extract *(*4μL/mL) did not significantly reduce the pSTAT-1/STAT-1 ratio, while the three higher doses did reduce pSTAT-1/STAT-1 levels. The highest dose (9.0μL/mL) elicited a 76% reduction in pSTAT-1/STAT-1 based on the calculated band densitometry (Fig 3, P < 0.05).

### Fennel seed extract exerts protective effects on barrier integrity in DSS colitis

**Fig 4:**
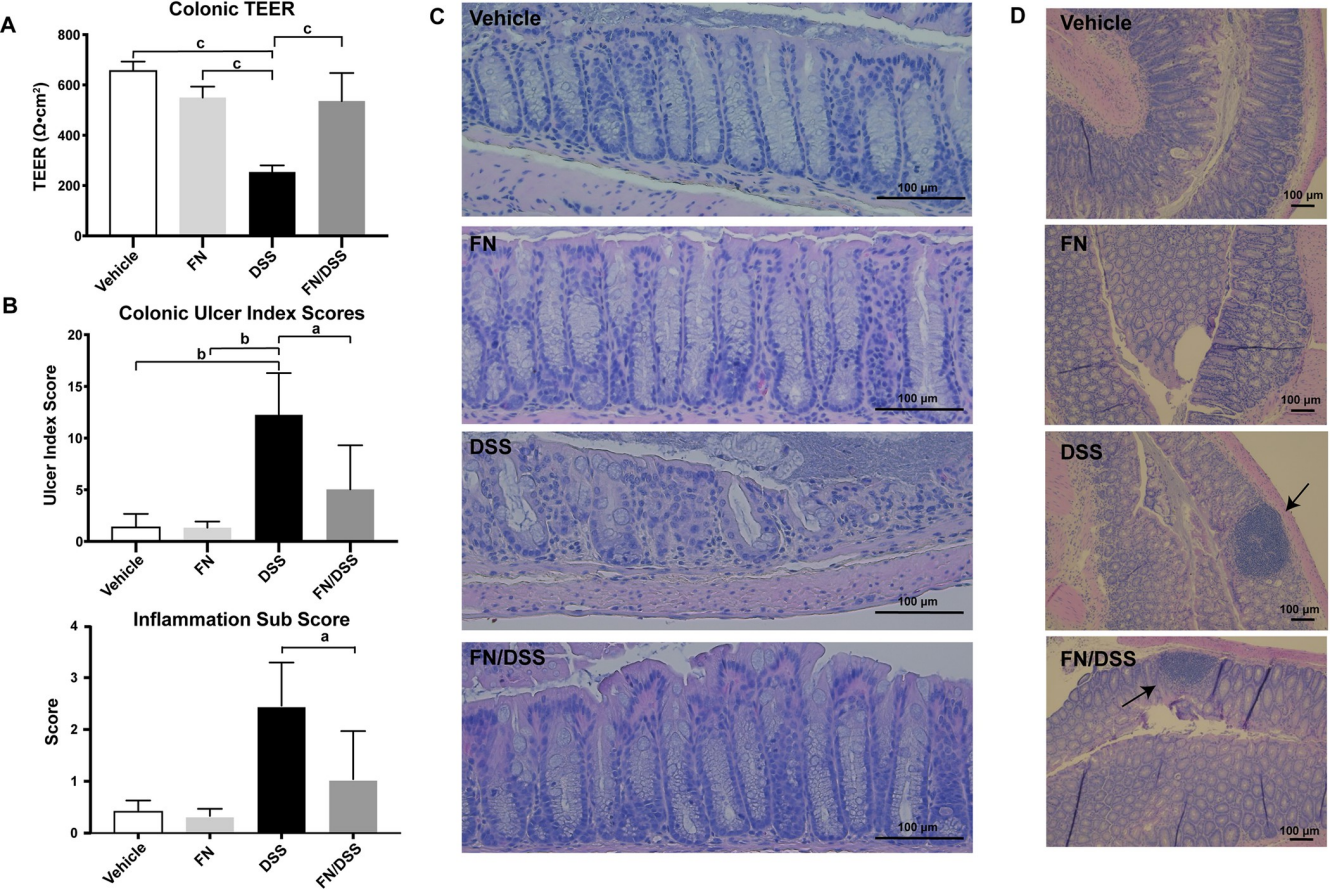
Fennel seed extract has beneficial effects on barrier integrity in mice with DSS colitis. A) fennel seed extract (FN) prevents loss of TEER in FN/DSS treated mice compared to DSS treated mice. B) fennel seed extract (FN) treated mice show improved ulcer indices compared to DSS treated mice. Inflammation sub score also shown, mirror improvement in overall ulcer indices. C) Representative H&E staining of mid colons of treated mice showing reduced inflammation, ulceration and architectural changes in fennel seed extract (FN) treated mice. D) Representative H&E staining of mid colon demonstrating inflammation in DSS and Fennel seed extract/DSS treated mice. N = 4; Fig 3C: 20x magnification, Fig 3D: 10x magnification; a = P < 0.05; c = P < 0.001.

To investigate whether our findings were relevant *in vivo*, parameters of colitis and barrier integrity were measured in mouse intestinal tissues following induction of colitis in the presence or absence of treatment with fennel seed extract. Upon sacrifice, 3 of 4 DSS treated mice presented with bloody stool, while the mice treated with fennel seed extract had softer and lighter colored stool samples. Colonic tissue obtained from DSS treated mice was also more friable, or easier to damage through manipulation. Fennel seed extract treated and control mice demonstrated normal colonic integrity when handled. The TEER across mid colonic tissues of fennel seed extract treated mice showed significantly higher values than DSS treated mice (Fig 4A, n = 4). There was no observed difference among control mice (vehicle treated) and fennel seed extract treated mice.

Histological analysis of colonic mucosa revealed decreases in crypt distortion, depth of ulceration and inflammatory infiltrates in DSS/ fennel seed extract mice vs. DSS mice while the histologic appearance of colons from control and fennel seed extract groups were normal. Inflammation subscores confirmed the potency of DSS treatment, showing a significant inflammatory response in DSS treated mice and a lesser response in fennel seed extract /DSS treated mice (Fig 4B and 4D). Ulceration was present in 50% of DSS mice, with a representative ulcer shown (Fig 4D). Ulcer indices were decreased in fennel seed extract */*DSS mice vs DSS mice (Fig 4B and 4C, P < 0.001).

### Fennel seed extract attenuates activation of STAT1 in DSS mice

**Fig 5:**
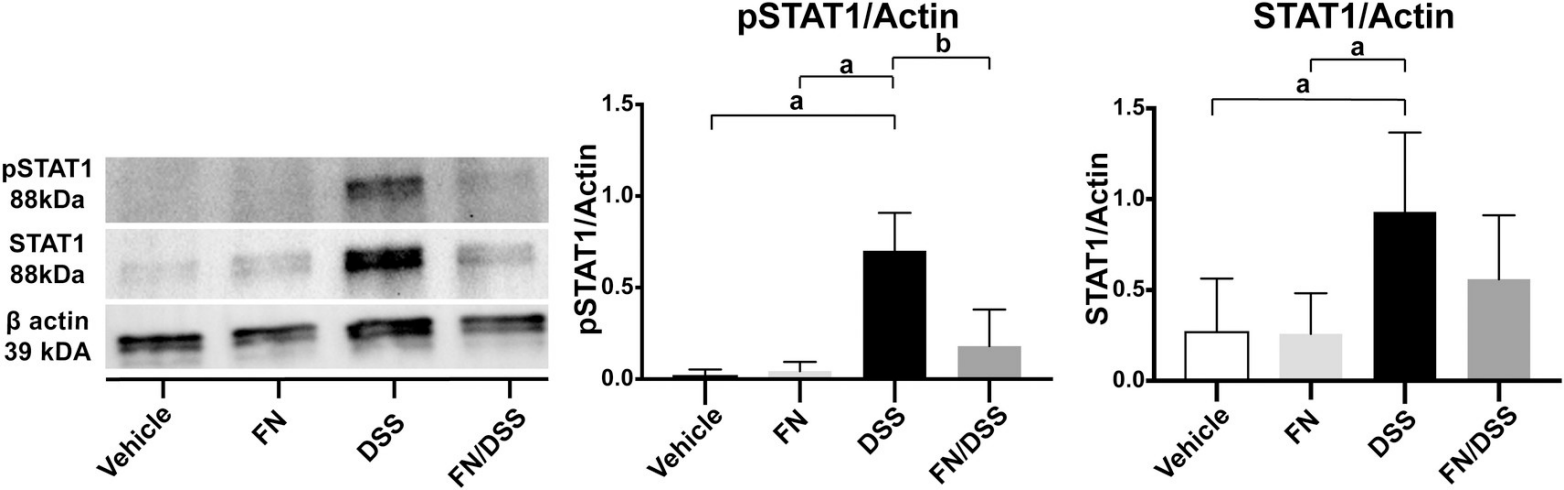
Fennel seed extract reduces protein expression of pSTAT1 in mice with DSS colitis. Western blot analysis demonstrates significantly reduced pSTAT1 in FN/DSS mice compared to DSS mice. Quantification of band density shown. pSTAT1 and STAT1 density adjusted for β-actin levels. Data presented as mean + upper SD. N = 4; a = P < 0.05; c = P < 0.001.

The level of pSTAT1 and total STAT1 protein was measured in mouse colonic tissues from control, fennel seed extract, DSS and fennel seed extract */*DSS groups. Western blot analysis showed that fennel seed extract/DSS treated mice had significantly decreased levels of pSTAT1 compared to DSS treated mice (Fig 5). As expected, minimal pSTAT1 was detected in control and fennel seed extract mice, indicating no active inflammatory processes (Fig 5). Treatment with DSS increased levels of total STAT1 compared to controls and fennel seed extract did not significantly attenuate this increase (Fig 5). pSTAT1 and STAT1 levels were compared to actin levels due to changes in membrane background preventing direct comparison of pSTAT1 to STAT1 band density.

## Discussion

In this study, an ethanolic extract of fennel seed was shown to downregulate STAT1 activation and improve gastrointestinal barrier function in both *in vitro* and *in vivo* models. Fennel seeds are known to contain volatile compounds and odorants including biologically active anethole [41, 42]. Previous studies have shown that *F*. *vulgare* has anti-oxidant effects and it is used in some areas of the world as a complementary and alternative treatment for IBD, but the effect of fennel on barrier function and the JAK/STAT pathway, a major contributor to IBD pathogenesis, had not previously been examined [9]. The current medical treatment of IBD includes aminosalicylates, glucocorticosteroids, immunosuppressants, and biological therapies which all have side effects ranging from mild to life-threatening [43, 44]. Due to the common side effects of current IBD treatments, alternative therapies, such as *F*. *vulgare*, have potential adjunctive roles.

This work investigated the effect of fennel seed extract on tight junction proteins in the intestinal epithelium. The T84 cell line was used as a simple *in vitro* model reflective of colonic epithelial properties and treated with IFN-γ to model inflammation. T84 cells that were pre-treated with fennel seed extract prior to IFN-γ treatment displayed significantly increased transcription of OCLD and TJP-1 compared to controls that did not receive pre-treatment. Most notably, increases in transcription of TJP-1 showed a dosage effect with increased transcription seen as the dose of fennel seed extract was increased. OCLD and TJP-1 are involved in regulating tissue electrical resistance, evidenced previously by OCLD-dependent increases in TEER [45]. Increased TEER and improved cell-cell interactions have been linked, which is largely due to strengthening of the zonula occludens junction, where OCLD and TJP-1 are located. Epithelia with high TEER have been shown to have zonula occludens with more strands compared to epithelia with low TEER [33]. The increases in OCLD and TJP-1 transcripts provide a potential explanation for the significantly improved TEER under inflammatory conditions. Interestingly, our mRNA data did not show the expected decreases in OCLD nor TJP-1 mRNA in the IFN-γ only treatment group that have been established in previous literature [46]. Instead, similar levels of OCLD and TJP-1 mRNA were noted between the IFN-γ treated and control cells. On the other hand our staining data showed mislocalization of tight junction proteins, revealing the detrimental effect of IFN-γ on the cells as reported earlier [46]. Hence, the absent tight junction modulation is not attributed to lack of response to IFN-γ challenge, as also evidenced by the increase in pSTAT1 levels. The redistribution/mislocalization of the tight junction proteins into the cytosol supports previous studies that have shown tight junction regulation can be controlled via intracellular trafficking of tight junction associated proteins. TNF cytokines have been shown to induce calveolin-1 mediated endocytosis of occludin and restructuring of ZO-1 structures [47, 48]. Therefore, in our studies, tight junction modulation was likely occurring at the level of protein (intracellular trafficking) and not at the level of gene transcription. Our data suggest that fennel seed extract attenuates the reduction of important barrier proteins in a model of the inflammatory state *in vitro*, with evidence of similar effects in the *in vivo* murine model. Studies also revealed several mechanisms for cytokines to disrupt the apical junction complex, including by affecting the perijunctional cytoskeleton via myosin regulatory light chain [49, 50], disrupting apical actin [46], and disrupting lipid composition [51]. It has also been reported that the proapoptotic effect of cytokines is independent of their influence on the epithelial junction complex [52].

In the DSS colitis mouse model, fennel seed extract was shown to ameliorate structural pathology and protect against DSS-induced TEER loss. DSS-induced colitis has previously been shown to involve macroscopic damage such as multifocal gland dilation, epithelial erosion and infiltration of inflammatory cells into the lamina propria [39, 53, 54]. In this study, the mid colon of mice was analyzed for the severity of ulcers and tissue damage. A significantly decreased ulcer index was found in the mid colon from Fennel seed extract/DSS mice compared to DSS mice. Additionally, our finding that fennel seed extract treatment prevented loss of TEER in DSS mice also presents evidence pointing toward an ameliorative role. Thus, we conclude that fennel seed extract mitigates colitis symptoms at both the macroscopic and histological level.

**Fig 6:**
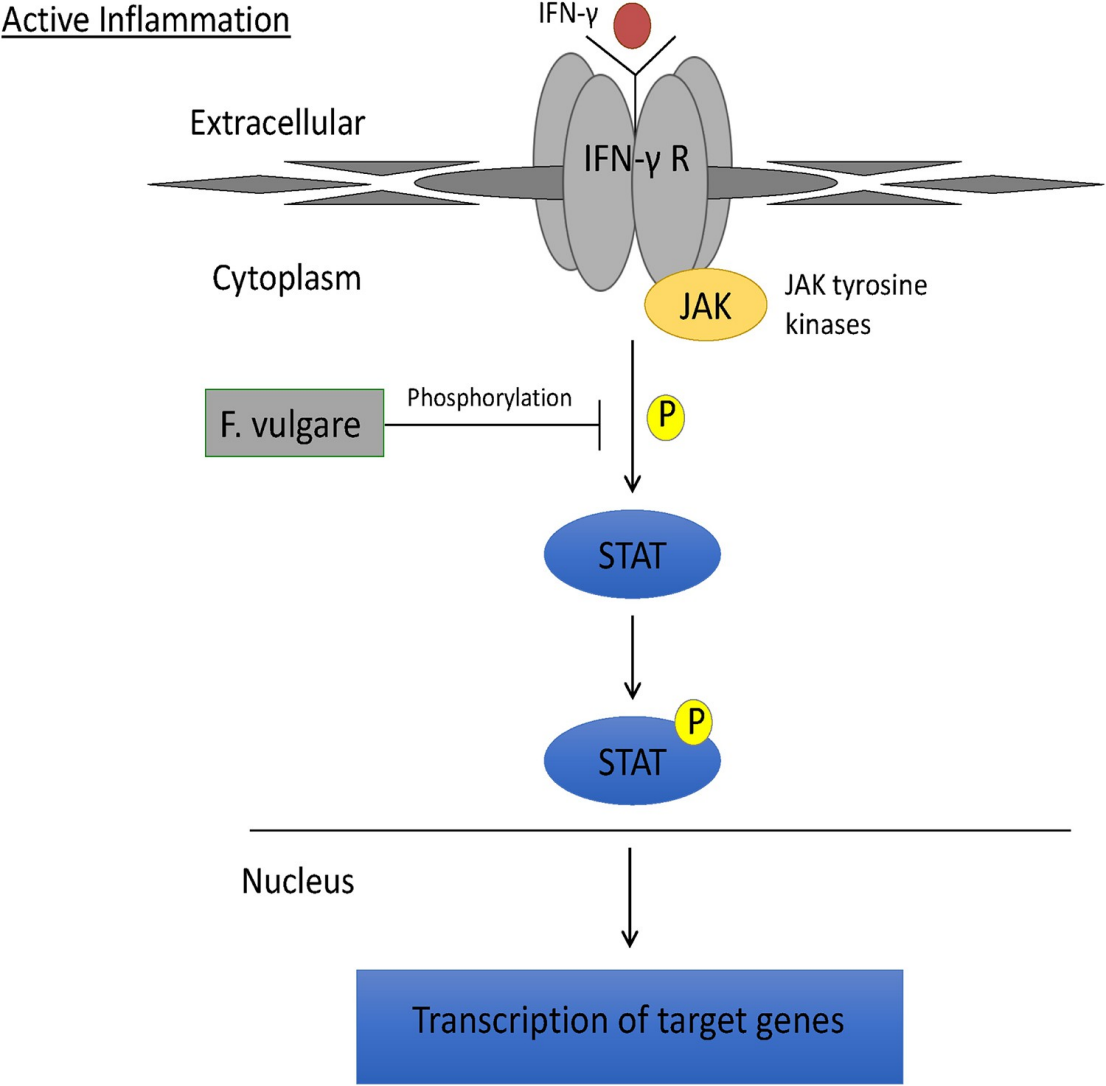
Schematic representation of mechanism of STAT pathway attenuation in T84 model. Fennel seed extract prevents phosphorylation of STAT1, preventing transcription of other inflammatory genes.

Based on the protective effect of fennel seed extract on barrier function and integrity during inflammation, the JAK/STAT pathway was investigated. Western blotting demonstrated levels of total STAT1 to be similar across experimental conditions. STAT1 is a major mediator in the JAK/STAT pathway leading to downstream transcription of inflammatory gene targets [55]. Increased protein expression of STAT1 has been seen in ulcerative colitis in humans [56]. Despite this, fennel seed extract significantly attenuated the ability of IFN-γ to elevate pSTAT1 relative to total STAT1levels, suggesting that STAT1 activation and downstream events were impaired (Fig 6).

Although the exact mechanism by which fennel seed extract alleviates the negative effect of IFN-gamma treated cells or DSS induced colitis through modulating the JAK/STAT pathway unclear, many effects could be at play. Since the STAT1 phosphorylation is reduced back to control level with fennel treatment when compared with the active inflammatory state, it is possible that active ingredients of fennel seed extract may inhibit the kinase activity of JAK to prevent STAT 1 phosphorylation. Volatile oils from fennel have been reported to inhibit neutrophilic inflammation through modulating phosphorylation of MAPK pathway [57]. Thus fennel seed extract may act similarly on the JAK/STAT pathway. Moreover, it is possible that fennel seed extract may inhibit IFN signaling by blocking its receptor. These nuances, though beyond the scope of this study, are worthy of investigation.

In murine mid colon, pSTAT1 was elevated when colitis was induced by DSS, as it is in the setting of human IBD. pSTAT1 was significantly decreased in fennel seed extract/DSS mice vs. mice that received DSS alone. Therefore, fennel seed extract likely reduces activation of STAT1 primarily by inhibiting its phosphorylation, as it did *in vitro* (Fig 4). We note that there was also a nonsignificant decrease in total STAT1 relative to actin in fennel seed extract/DSS mice vs. DSS mice. Thus, comparing levels of pSTAT1/STAT1 as a ratio appeared to mask the absolute level of inflammatory response generated within the mice. Importantly, mice treated with only fennel seed extract had similar expression of STAT1 to that in controls. This suggests fennel seed extract plays a role in the state of active inflammation *in vivo*, inhibiting function of STAT1 only when mice are exposed to DSS. This may be similar to the known protection of epithelial barrier function by the Crohn’s disease associated gene protein tyrosine phosphatase [58]. Additionally, STAT phosphorylation has been shown to decrease TEER via Claudin-2 induction and prevent expression of adipogenesis related genes, both of which were also shown to be reversible via JAK inhibitor treatment, thereby reducing STAT phosphorylation [59, 60].

Due to the success of fennel seed extract in mitigating inflammatory response in both models of IBD, fennel appears to be a promising candidate for further clinical trials to measure its efficacy as a complementary or alternative therapeutic. The improvements in gut health seen in this manuscript and other manuscripts examining herbal remedies lend credence to the notion of many undiscovered remedies or therapies for other chronic diseases. Identification of the active component(s) within fennel seed extract was not explored in this study, limiting our understanding of the exact mechanisms of action. Future studies could pursue this knowledge, which would yield a more controllable therapeutic. The human equivalent dosage of fennel seed extract given to mice in this study is 146 mg/kg; therefore interested clinical trials could consider starting dosages of 14.6 mg/kg [61]. Extract could be delivered in a similar way as given to mice (using water as vehicle) or simply added to food until the target dosage is reached.

## Conclusion

Our findings regarding fennel seed extract and its effect on the STAT pathway show parallels to current therapeutic approaches for IBD. Systemic glucocorticoids, often used to induce remission of IBD, lead to decreased levels of pSTAT1 in mucosal samples of patients with ulcerative colitis [56]. A novel JAK inhibitor, tofacitinib, was recently shown to induce clinical remission of UC in a randomized phase 3 trial [62, 63]. The treatment shown here with fennel seed extract is in line with these IBD treatment modalities as all three target the JAK/STAT pathway to critically reduce inflammatory signaling in the intestines. The active ingredients of fennel seed have been reported to have anti-inflammatory effects and protective effects against injury in different studies [57, 64, 65]. Due to its beneficial effect, different forms of fennel are included in specific diets to improve the symptoms of IBD [10, 66, 67].

Due to the protective role of fennel seed extract on barrier function and inflammatory proteins, fennel seed extract has a potential role in IBD treatment. Fennel seed extract reduced histological and functional damage in mice with DSS-induced colitis. Fennel seed extract was also shown to have an anti-inflammatory effect associated with reduced activation of the STAT pathway in mice with DSS-induced colitis and in intestinal epithelial cells. As therapeutics targeting JAK/STAT activity are showing promise in the treatment of IBD, this further underscores the potential for fennel seed extract in combatting disease manifestations. Additionally, such studies lend credence to the notion of consuming fennel seeds for digestive health.

## Supporting information

S1 Raw images (PDF)

## Abbreviations

IBD: inflammatory bowel disease
IBS: irritable bowel syndrome
HE: hematoxylin and eosin
JAK: Janus Kinase
STAT: Signal Transducer and Activator of Transcription
IFN- γ: interferon-gamma
pSTAT1: phosphorylated STAT1
*F. vulgare*: *Foeniculum vulgare*
TJP-1: tight junction protein-1
DSS: dextran sodium sulfate
TEER: transepithelial electrical resistance
