# Supplementary figures and images for: The effect of a fennel seed extract on the STAT signaling and intestinal barrier function
Source: PLoS One. 2022 Jul 8;17(7):e0271045. doi: 10.1371/journal.pone.0271045 (PMC9269469; doi:10.1371/journal.pone.0271045)

Control  
IFN  
FN 4ug/ml+ IFN  
FN 6ug/ml+ IFN  
FN 7.5ug/ml+ IFN  
FN 9ug/ml+ IFN

**pSTAT 1**

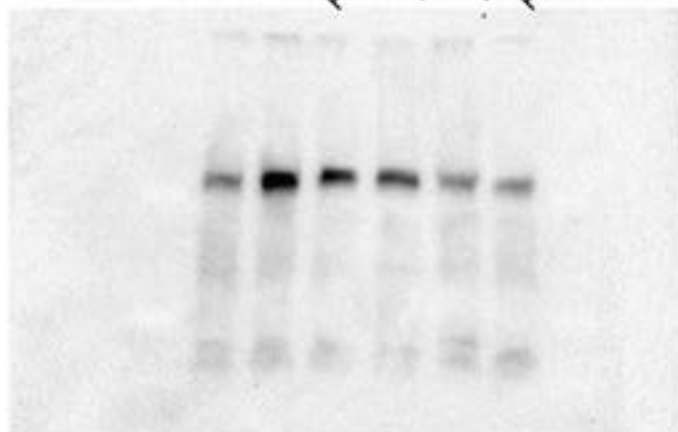

**STAT 1**

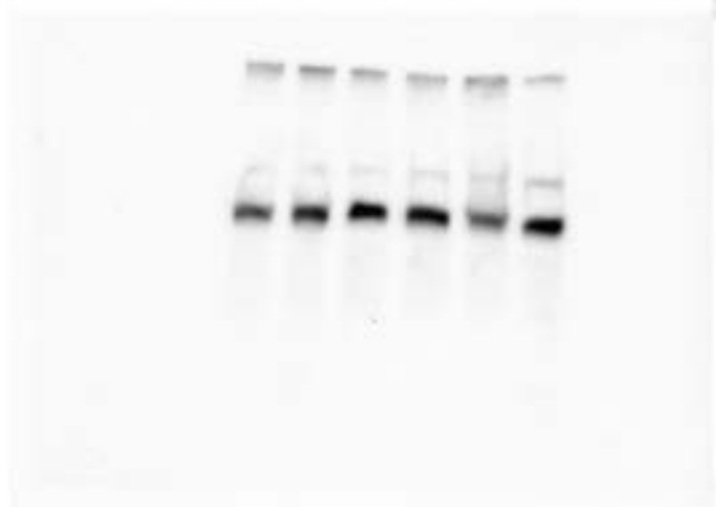

**Actin**

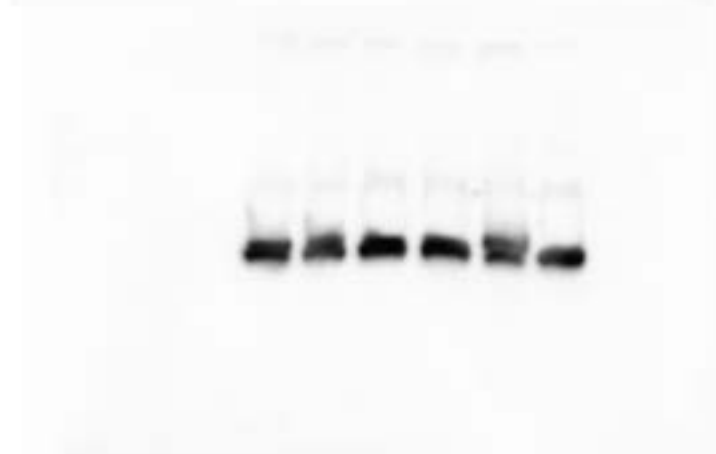

**pSTAT 1**

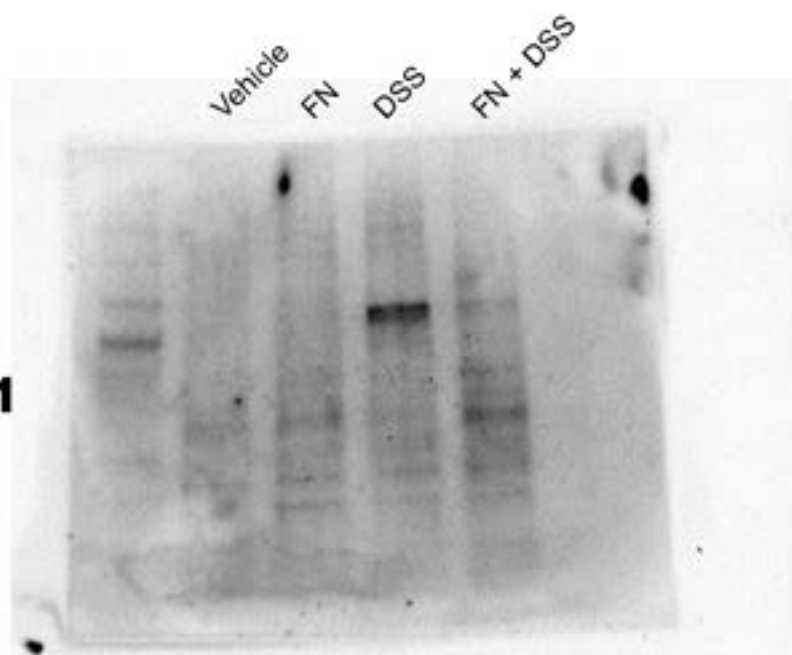

**STAT 1**

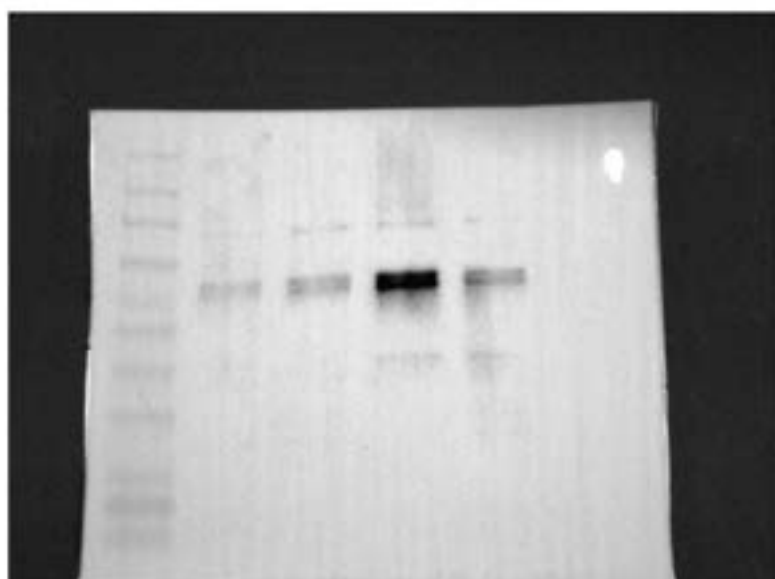

**Actin**

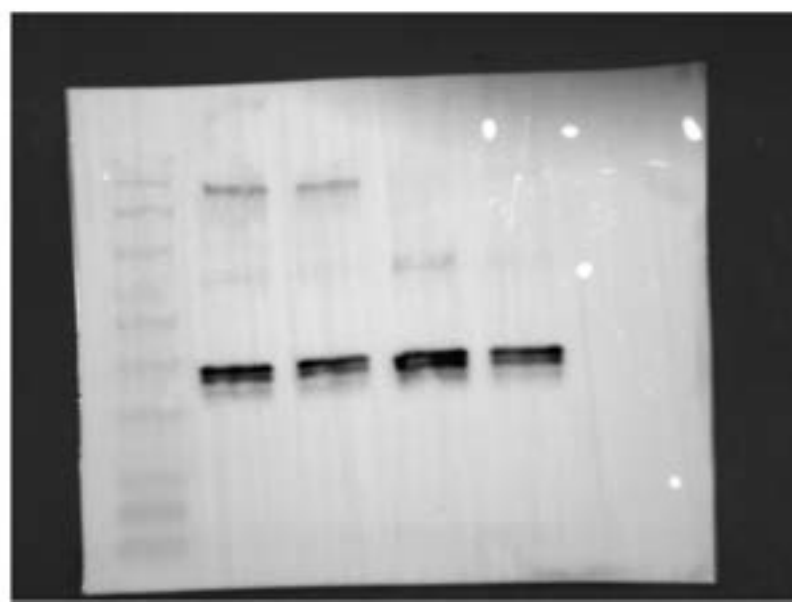

Supplement: S1 Raw images — (PDF) [file pone.0271045.s001.pdf]
